# The context and mechanisms that contribute to accessible and engaging asthma care: a rapid realist review

**DOI:** 10.1183/16000617.0042-2026

**Published:** 2026-11-04

**Authors:** Emma Kinley, Hilary Pinnock, Huayi Huang, Jessica Sheringham, David Lo, Irtiza Qureshi, Tracy Jackson, Jennifer Quint, Constantinos Kallis, Gwyneth A. Davies, Erol A. Gaillard, Purva Abhyankar

**Affiliations:** 1 School of Psychology, Liverpool John Moore's University, Liverpool, UK; 2 Usher Institute, University of Edinburgh, Edinburgh, UK; 3 Research Department of Primary Care and Population Health, Institute of Epidemiology and Health Care, University College London, London, UK; 4 Department of Respiratory Sciences, University of Leicester, Leicester, UK; 5 Centre for Inequalities, Institute for Lifecourse Development, University of Greenwich, Greenwich, UK; 6 School of Public Health, Imperial College London, London, UK; 7 Department of Population Data Science, Swansea University Medical School, Swansea, UK

## Abstract

**Introduction:**

Access to and engagement with asthma care could improve outcomes, but there is limited understanding of the reasons for poor engagement and a gap in actionable evidence to inform how to reduce inequities. We aimed to understand the context, mechanisms and outcomes (CMOs) that affect access to and engagement with asthma care and to identify solutions.

**Methods:**

Using rapid realist review methodology, we searched four databases (1999–2024) for studies of any design in asthma populations (>5 years of age) that explored access or engagement in healthcare applicable to the UK health context. We extracted CMO configurations explaining access and engagement with asthma services and synthesised them into overarching theories. External reference group meetings with 16 experts with lived experience and professional expertise were held to discuss preliminary review findings and inform their interpretation.

**Results:**

19 articles reported 16 studies. We identified eight theories from 51 CMOs, grouped under two themes. 1) Demographic and social–cognitive barriers, including socioeconomic and structural barriers, uncertain candidacy resulting in escalation to emergency care, low perceived need hindering engagement and relational accountability driving engagement. 2) Health system barriers, including over-burdened services, fragmented systems disrupting care, feeling unheard hindering youth engagement, and cultural and language barriers. Solutions included digital care, education, developing patient skills, proactive follow-up, training for healthcare professionals, information sharing between services and enhanced professional–patient relationships.

**Conclusion:**

Enhancing system coordination, cultural sensitivity and patient-centredness of care may improve trust and patient–provider communication, leading to better engagement and proactive asthma care.

## Shareable abstract

**Healthcare professionals need to be proactive in how they approach engaging patients for asthma care, increase the perceived value of consultations and tackle systemic barriers and reduce fragmentation to increase access and engagement with asthma care.** https://bit.ly/4xRRqax

## Introduction

Asthma is a common chronic condition affecting all ages and all ethnic groups [1]. The disease burden arises from persistent symptoms that impair the quality of life of individuals and their families. Asthma attacks can disrupt education and work, require urgent access to healthcare, and can be fatal. In the UK, 5.4 million people live with asthma [2], resulting in over 6 million primary care consultations, nearly 100 000 hospital admissions and over 1000 deaths every year (including 20 deaths in children under 14 years) [3], at an annual cost to the National Health Service (NHS) in excess of £1 billion [3]. Each year, 29% of adults with asthma are prescribed a course of oral steroids and 4% are admitted to hospital [4]. There is a clear link between socioeconomic deprivation and poor lung health, with more than half of children who die from an asthma attack coming from the most deprived communities [5]. These UK asthma outcomes are amongst the worst in Europe [6, 7].

For three decades, national and global guidelines have recommended evidence-based interventions to reduce this burden [1, 8, 9]; however, these interventions often fail to reach those who need them. Only a third of people with asthma in the UK receive “basic care” (an annual asthma review including provision of supported self-management) [10]. Regular use of inhaled corticosteroids improves asthma control and reduces the risk of attacks, but fewer than half of adults and children with asthma collect the prescribed controller medication needed to adhere to recommended regimes [11, 12]. Nearly three quarters of people with severe asthma who could benefit from biologic treatment remain “hidden” in primary care, missing out on highly effective treatments [13, 14]. A quarter of asthma patients who had an attack recorded in the previous year did not attend an asthma review in primary care within the following 12 months [15].

Some individuals and groups (*e.g.* those who are socioeconomic disadvantaged) face greater barriers to accessing or engaging in asthma care and have worse asthma outcomes (*e.g.* increased exacerbations) [16]. Mechanisms for such inequalities are multi-faceted, encompassing poor implementation (*i.e.* interventions not accessible in routine practice) and poor uptake (*i.e.* patients not engaging with available care). How people with asthma manage their condition is shaped by their personal experiences, individual (and family) health beliefs, cultural background and socioeconomic circumstances, as well as practical factors relating to their local context, environment and domestic or work arrangements [17, 18]. Importantly, patients’ ability to access and engage with evidence-based care is affected by local professional, organisational and contextual factors, as well as upstream policy.

A comprehensive understanding of the multi-level factors influencing access to and engagement with asthma care is required to develop and implement service-level interventions [19]. Within a wider programme of work [20], we sought to achieve this understanding (in both adults and children 5 years and over) through a rapid realist review (RRR) of existing literature. We aimed to identify the barriers, mechanisms and contexts in which they operate, and to identify contextually relevant interventions likely to target mechanisms associated with the specific outcomes of improved access and engagement with asthma care.

### Key term

- CMO configuration: a CMO configuration explains how and why an intervention works (or not) by linking the context in which it is implemented, the mechanisms it triggers (the underlying processes or responses) and the resulting outcomes. It helps identify what works, for whom and under what circumstances.

## Methods

### Study design

Realist review methodology is a theory-driven approach to reviewing the literature that seeks to understand why certain outcome patterns, such as good or poor access to and engagement with asthma services, occur in health systems [21, 22]. The purpose of the review is explanatory, *i.e.* to produce one or more theories to explain particular phenomena [23]. Theorising in realist approach consists of identifying CMO configurations that explain how specific circumstances (context) trigger or activate underlying processes (mechanisms) to produce certain results (outcomes or phenomena). The concept of “mechanism” is central to realist reviews. Mechanisms are underlying causal entities or processes (people's reasoning, preferences, norms, beliefs, reactions and behavioural responses) that operate in particular contexts to generate observed outcomes [23]. In this review, we adopt the RRR process developed by Saul *et al.* [22].

This method applies a resource-efficient realist approach to produce timely knowledge synthesis for policymakers responding to emerging issues. This RRR is reported in line with the RAMESES standards for realist syntheses [24]. The review protocol was registered in PROSPERO (CRD42024534978).

### External reference group and patient and public involvement

An integral component of an RRR is engagement with an external reference group (ERG) comprising experts who contribute to data identification, support theory development, ensure that the review remains grounded in the local context and fill any gaps in the published literature [21, 22]. For this review, we convened two groups: 1) a multidisciplinary group comprising researchers and clinicians (primary and secondary care practitioners, adult and paediatric nurses, pharmacists, general practitioners, paediatricians, respiratory specialists, severe asthma specialists, integrated care specialists and primary care respiratory specialists), as well as experts in public health, sociology, global inequalities and ethnic minority health; and 2) members of a patient and public involvement (PPI) group. PPI members had experienced barriers to accessing their asthma care and included people from underserved areas, people from minoritised ethnic communities and those who had experienced houselessness. Each group met separately on two occasions: initially to refine the project scope and assess the proposed full-text articles for inclusion, and subsequently to review the findings from data extraction and provide guidance on data synthesis. Their views on access and engagement with asthma care were discussed to ensure that our emerging theories aligned with current practice and patient experiences.

### Search process and selection and appraisal of documents

We searched PsycINFO, Embase, Ovid Medline and Cochrane Library in November 2024 for studies of any design that informed our understanding of barriers to accessing and/or engaging with healthcare and strategies to improve access and/or engagement among people with asthma (>5 yrs). We were guided by conceptual framework developed by Levesque et al. [25] in our consideration of access and our search for papers. Forward citation searching of key included papers was undertaken on 30 January 2026 *via* Web of Science to identify subsequent relevant publications; however, no additional articles were found. Key search terms can be found within supplementary material 1. Using the PICO (population, intervention, comparator and outcome) framework [26], eligibility criteria were developed (table 1). Studies identified outside of the systematic search process (*e.g.* including grey literature and relevant literature identified by the ERG) were checked against the eligibility criteria and included within the review process. Records were selected in accordance with relevance to the programme theory (relevance, rigour and richness) and inclusion and exclusion criteria. Title and abstracts were screened independently by team members H. Huang, D. Lo, E. Kinley, I. Qureshi, E.A. Gaillard, H. Pinnock and J. Sheringham (listed in order of screening contribution) using Covidence (Veritas Health Innovation, Melbourne, Australia; www.covidence.org). All disagreements were resolved by team review and discussion.

**TABLE 1:** Inclusion and exclusion criteria following the PICO (population, intervention, comparator and outcome) framework [26]

	Inclusion	Exclusion
**Population**	Diagnosed or “highly suspected” asthma (*e.g.* referral for further asthma investigations) Adults and children 5 years and over (pre-school wheeze presents a diagnostic challenge and requires a different management approach [27, 28])	Not an asthma study or <50% data for an asthma subgroup >50% of study population are <5 years
**Phenomena of interest**	Access to asthma healthcare/services (*e.g.* anything designed to increase engagement or exploring reasons for poor access)	Not investigating a healthcare intervention/service exploring access and engagement in asthma
**Context**	Any “asthma service” or “asthma care” (any primary/secondary/tertiary healthcare) High- or middle-income countries	Not comparable to a UK socioeconomic or clinical context (we excluded studies based in countries with low healthcare expenditure (typically low- and middle-income countries), weak universal coverage, financial barriers to care, weak primary care and chronic disease management)
**Study design**	Primary or secondary empirical research: qualitative, quantitative (randomised controlled studies, before-and-after studies), observational, mixed-method, systematic/meta/realist reviews, *etc*. Other sources, including reports, test/opinion papers, book chapters, websites and grey literature	Conference abstract(s) (and no closely related full-text paper)

### Data extraction and synthesis

A bespoke data extraction form collected information on study characteristics (supplementary material 2) and the CMO configurations derived from the study's findings relating to access and/or engagement with asthma services. Initial data extraction was performed by E. Kinley and H. Huang, and the initial CMO configurations identified were then reviewed and refined iteratively by P. Abhyankar. CMO configurations were extracted from each study by working backwards from the reported outcomes relating to access to and/or engagement with asthma care or services (*e.g.* attendance or nonattendance at asthma reviews or follow-up, appropriate/inappropriate use of emergency or unscheduled care, and engagement with or disengagement from care) to identify when and for whom these outcomes occurred (study or clinical contexts), as well as why and how (mechanisms).

Data synthesis involved grouping individual CMOs according to similarities in contexts and/or mechanisms linked to the outcome of interest to develop overarching theories. P. Abhyankar and E. Kinley undertook this grouping based on evidence drawn directly from the included papers, identifying thematic threads that clustered around a central idea or hypothesis. Once individual CMOs were grouped into clusters, the explanatory theories within each cluster were written in full, revised and refined through iterative consultation with the study team. The developed theories were further grouped under two overarching themes reflecting contexts and mechanisms operating at different levels.

Due to the time constraints of an RRR, quality is addressed by involving an ERG comprising experts. We invited members of the expert panel to reconvene after the data extraction and synthesis phase. ERG feedback was incorporated into theory development to ensure that the final findings and themes addressed any gaps in practice that the analysis had not captured.

## Results

### Study selection and characteristics

The initial search was undertaken on 14 November 2024 and identified 2220 articles, of which 16 met the inclusion criteria and were included in the review. The PRISMA (Preferred Reporting Items for Systematic reviews and Meta-Analyses) flow diagram [29] shown in figure 1 provides details of the screening process. The 16 studies were reported in 19 articles (three articles were part of a larger programme of work and were combined into one overall study), all of which contributed data to the theories.

**FIGURE 1:**
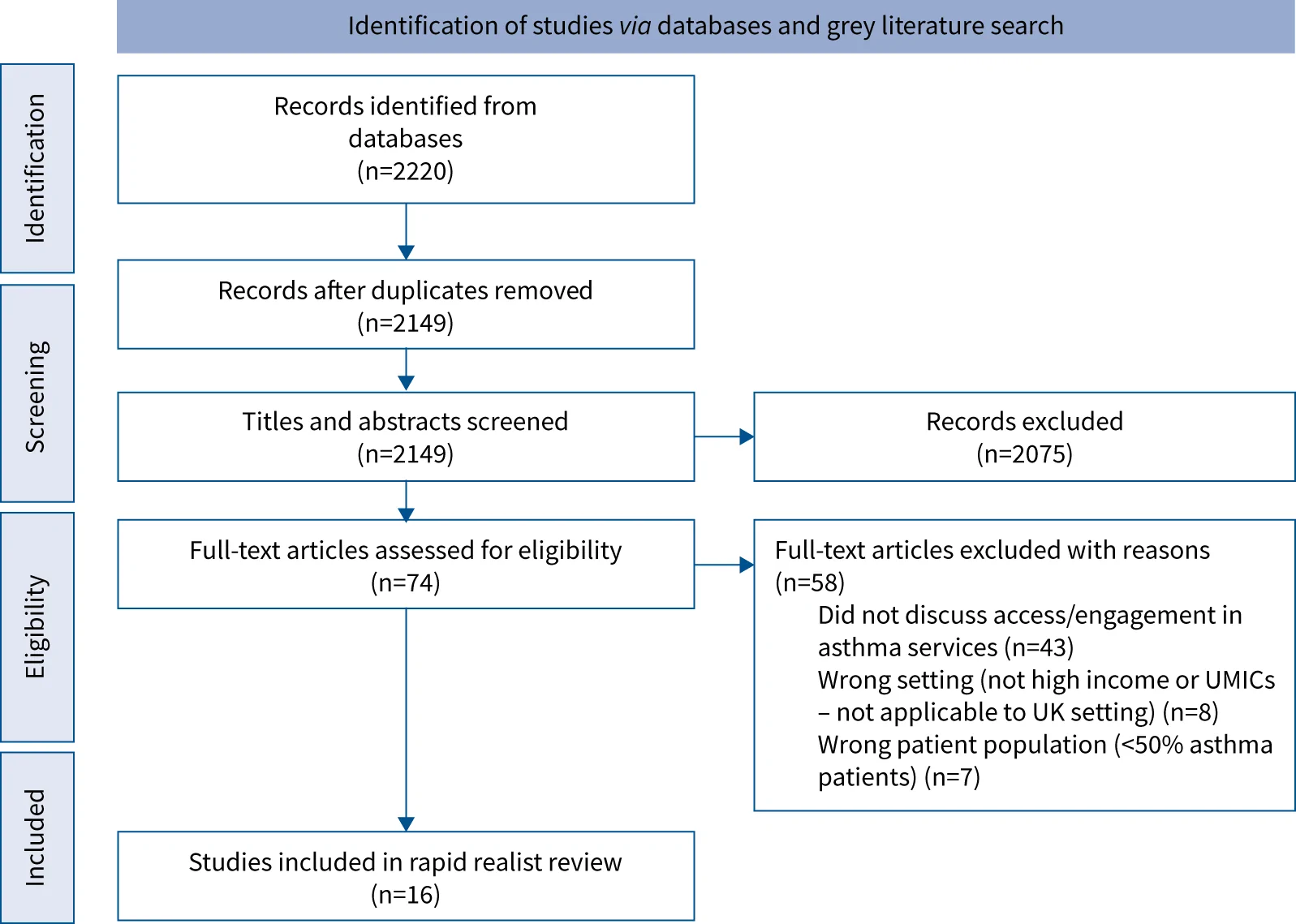
PRISMA (Preferred Reporting Items for Systematic reviews and Meta-Analyses) flow diagram [29]. UMIC: upper middle-income country.

All included studies were published between 1999 and 2024 and were undertaken in the UK (n=9), the USA (n=4) and Australia/New Zealand (n=2). One study was a systematic review of studies conducted in the UK, USA, Australia, Germany, Sweden and the Netherlands. Seven studies used qualitative methods and nine used quantitative methods. Six were published before 2010. Seven explored patients’ views, two explored providers’ views and one explored both. Six were observational studies of patient records or consultations with clinicians. Detailed study characteristics and extracted CMOs from the included studies can be found in supplementary material 2. Supplementary material 3 provides the theories developed from 16 studies (19 articles) explaining access and engagement with asthma services.

### Explanatory theories: what are the contexts and mechanisms that make people more or less engaged in asthma care?

A total of 54 explanatory CMO configurations extracted from included articles were synthesised into eight distinct theories, which were further grouped under two overarching themes reflecting contexts and mechanisms operating at different levels.

### Theme 1: demographic and social–cognitive barriers

This theme includes contexts and mechanisms operating at the individual and interpersonal levels (micro) that help or hinder access to and engagement in asthma care (see figure 2).

**FIGURE 2:**
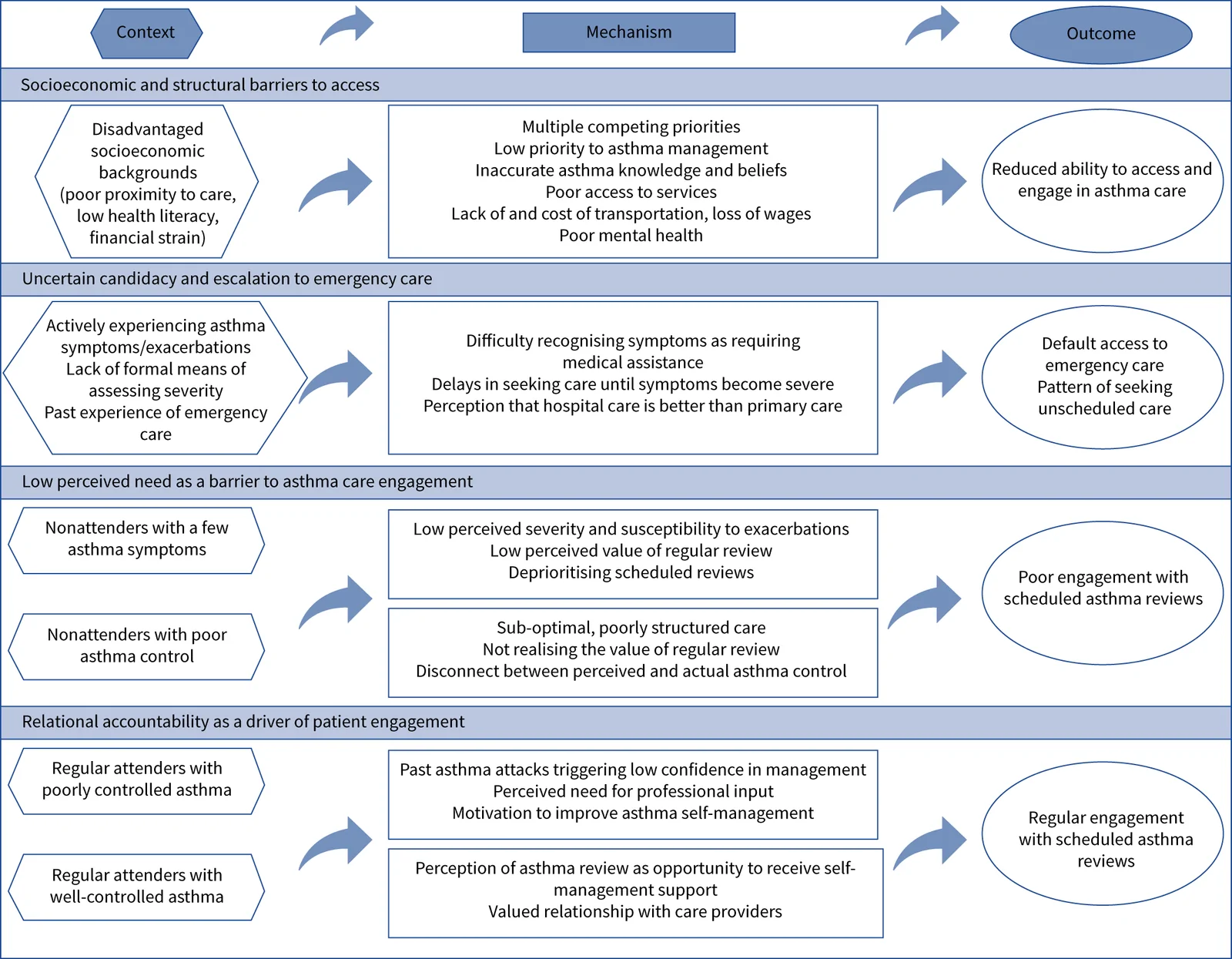
Demographic and social–cognitive barriers.

#### Theory 1: socioeconomic and structural barriers to access

*Disadvantaged socioeconomic circumstances, such as financial strain and uncertain or difficult employment circumstances (context), can mean that patients have more competing demands and greater constraints on their time and resources (mechanisms), resulting in a limited capacity to access care (outcomes).*

Five articles [30–34] explored several mechanisms that explain how and why socioeconomic disadvantage may lead to poor access and engagement. Hoffman *et al*. [32] found that nonengagement among these groups is due to limited and unaffordable transport, loss of earnings from missing work, and out-of-pocket healthcare costs, such as consultation and prescription fees. Distance from appropriate services was reported as a barrier to accessing scheduled care by Teach *et al*. [33] and Serhal *et al*. [34]. Fawcett *et al*. [31] found that children from disadvantaged and minoritised ethnic backgrounds were less likely to access care because their families often had competing priorities or routines, with children's asthma receiving lower priority than other family worries. Lower levels of health literacy were reported by Buetow *et al*. [30] as a barrier to engagement when patients held inaccurate beliefs about asthma and underestimated the severity of disease. ERG members added poor mental health and other comorbidities as additional mechanisms that may create barriers for people from lower socioeconomic backgrounds in systems that rely on patients’ initiative to access or engage in proactive care.

*Telephone asthma review consultations (intervention) can improve access to routine care (outcome) by overcoming structural barriers to attendance (mechanism)*.

Interventions that target contextual barriers (*e.g.* difficulties attending appointments faced by people on low incomes) or mechanisms (*e.g.* reducing the costs, time and absence from work associated with access to asthma care) may improve engagement and access. Pinnock *et al*. [35] demonstrated that follow-up and scheduled asthma consultations can be delivered remotely by telephone and are effective in improving patients’ access without any clinical disadvantage or loss of satisfaction. Telephone reviews help overcome some of the structural barriers to access (*e.g.* by removing transport costs and the need to miss or reschedule work and other commitments, enabling families to prioritise asthma management) and were found to be acceptable and convenient modes of consultation by patients.

#### Theory 2: uncertain candidacy and escalation to emergency care

*Patients with asthma or parents of children with asthma who have active symptoms or exacerbations (context) often struggle to recognise when symptoms require medical attention, leading to delays in seeking care (mechanism) and defaulting to emergency services if symptoms become severe (outcome). This uncertainty around candidacy for care, compounded by a lack of formal means of assessing severity and past experiences of hospital care, reinforces patterns of emergency over routine care use (outcome).*

Six articles contributed data to the theory suggesting that “uncertain candidacy for healthcare” is a key causal mechanism underlying asthma exacerbations and attacks and subsequent patterns of repeated emergency care use. Hudson *et al*. [36] and Lakhanpaul *et al*. [37–39] found that, when actively experiencing asthma symptoms or exacerbations, patients or parents of children with asthma experienced difficulty in recognising when symptoms required medical assistance (uncertain candidacy), often due to a poor understanding of the causes and management of asthma and a lack of any formal means of assessing severity (*e.g.* action plans and peak flow meters). This led to delays in seeking help until symptoms became more severe and required urgent attention from emergency care, which was sought either directly or on the advice of emergency helplines. Similarly, Coffey *et al*. [40] found that families decide to access emergency care when they perceive their child's symptoms to be worsening or that their action plans are failing to work. In both studies, the experiences of being referred to and receiving emergency care often led to perceptions that care is better in hospitals as staff have more expertise and knowledge about asthma compared to primary care clinics, reinforcing a pattern of seeking unscheduled care [36–40].

*Timely follow-up after exacerbations (intervention) may increase patient receptiveness (mechanism), improving engagement (outcome).*

Theory 2 highlighted the need to provide education, skills and tools to help patients recognise worsening symptoms and affirm their candidacy for care, which is typically delivered through self-management education. However, patients’ nonengagement in routine asthma reviews, during which self-management education is provided, is a key barrier to preventing asthma exacerbations and improving control. Daines *et al*. [18] found that reviewing patients’ asthma immediately after emergency care for an exacerbation may make them more receptive to self-management support and lead to better engagement in asthma care.

#### Theory 3: low perceived need as a barrier to asthma care engagement

*Low perceived value of regular asthma reviews and a disconnect between perceived and actual asthma control (mechanism) among asthma patients (context) can lead to poor engagement with health services (outcome).*

Gruffydd-Jones *et al*. [41] found that 63% of nonattenders of structured nurse-led asthma care in UK general practice had low symptom morbidity and infrequent oral corticosteroid usage. Reasons for nonattendance included “asthma not serious enough to warrant a regular check-up” and “see own GP instead”, suggesting low perceived asthma severity and susceptibility to exacerbations and, consequently, low perceived need for regular care. Hoffman *et al*. [32] found that patients with mild or well-controlled asthma tended to deprioritise attending follow-up care for asthma to avoid adjusting their work schedules. However, approximately 50% of “low perceivers” in the study by Gruffydd-Jones *et al*. [41] had experienced asthma symptoms in the last month, and those who visited their general practitioner (GP) instead of the nurse-led asthma clinic exhibited significantly higher asthma morbidity, indicating poor control. The limited evidence of structured asthma care involving the use of action plans in patient records suggested that sub-optimal care, rather than higher illness severity, was responsible for poor control. This study emphasised the importance of formalised follow-up and self-management education for patients with seemingly good asthma control.

This theory was supported by the ERG, who agreed that nonattendance at regular reviews is often due to patients not realising the value of attending and viewing reviews as unhelpful, indicating the need to increase perceived value to patients and to emphasise the importance of regular reviews.

*Formalised follow-up through telephone reviews or pharmacist-initiated GP referrals (intervention) can enhance patient awareness and perceived value of care (mechanism), resulting in increased accessibility and more proactive management (outcome).*

Theory 3 highlighted the importance of formalised follow-up aimed at changing patients’ perceptions of their asthma severity, susceptibility to exacerbations and the value of regular professional review. Two interventions were described that increased the perceived value of care. Telephone asthma reviews were not only convenient but also enabled more goal-oriented discussions [35]. Serhal *et al*. [34] showed that pharmacist-initiated GP referrals improved uptake of asthma reviews, possibly by enhancing patients’ awareness of their susceptibility to asthma exacerbations. Pharmacist-initiated referrals led to more treatment changes by GPs, as pharmacist assessments were perceived by GPs as thorough and credible.

#### Theory 4: relational accountability as a driver of patient engagement

*Valued patient–provider relationships and experiences of supportive reviews (mechanism) promote engagement with care (outcome) among patients who understand the importance of self-management (context).*

Two articles [18, 42] contributed to the theory that regular attendance at asthma reviews is driven by patients’ motivation to improve self-management of their asthma. Among those with poorly controlled asthma, motivation may stem from past traumatic experiences, such as asthma attacks or exacerbations, which trigger feelings of low confidence in managing asthma and a need for professional input on self-management strategies. A combination of experiential learning of self-management and input from health professionals during asthma reviews over time motivated patients with well-controlled asthma to view reviews as opportunities to reinforce self-management skills and strategies, as well as benefit from recent therapeutical advances in asthma care. Importantly, patients who value their relationship with care providers do not wish to jeopardise this relationship through nonattendance. The ERG, however, also highlighted the opposite effect on patient engagement, whereby previous negative experiences of asthma care or services may act as a deterrent to accessing or engaging with future care.

*Follow-up contact from providers after a missed appointment (intervention) can convey genuine care or concern (mechanism), motivating future engagement (outcome).*

A common reason reported for nonattendance at asthma reviews is forgetfulness [43], even among motivated patients. McDonough and Mault [43] contacted patients within 1 week of their missed appointment, gauged asthma control using the Royal College of Physicians’ “three questions”, explored reasons for nonattendance in a nonconfrontational manner, and offered a further appointment. They found that contacting patients and asking about their nonattendance prompted them to attend future reviews by creating a sense of obligation, motivated by a desire to maintain their relationship with the care providers and a perception of genuine concern about their care.

### Theme 2: systemic barriers

This theme includes contexts and mechanisms operating at the health system level (meso and macro) that influence access and engagement in asthma care (see figure 3).

**FIGURE 3:**
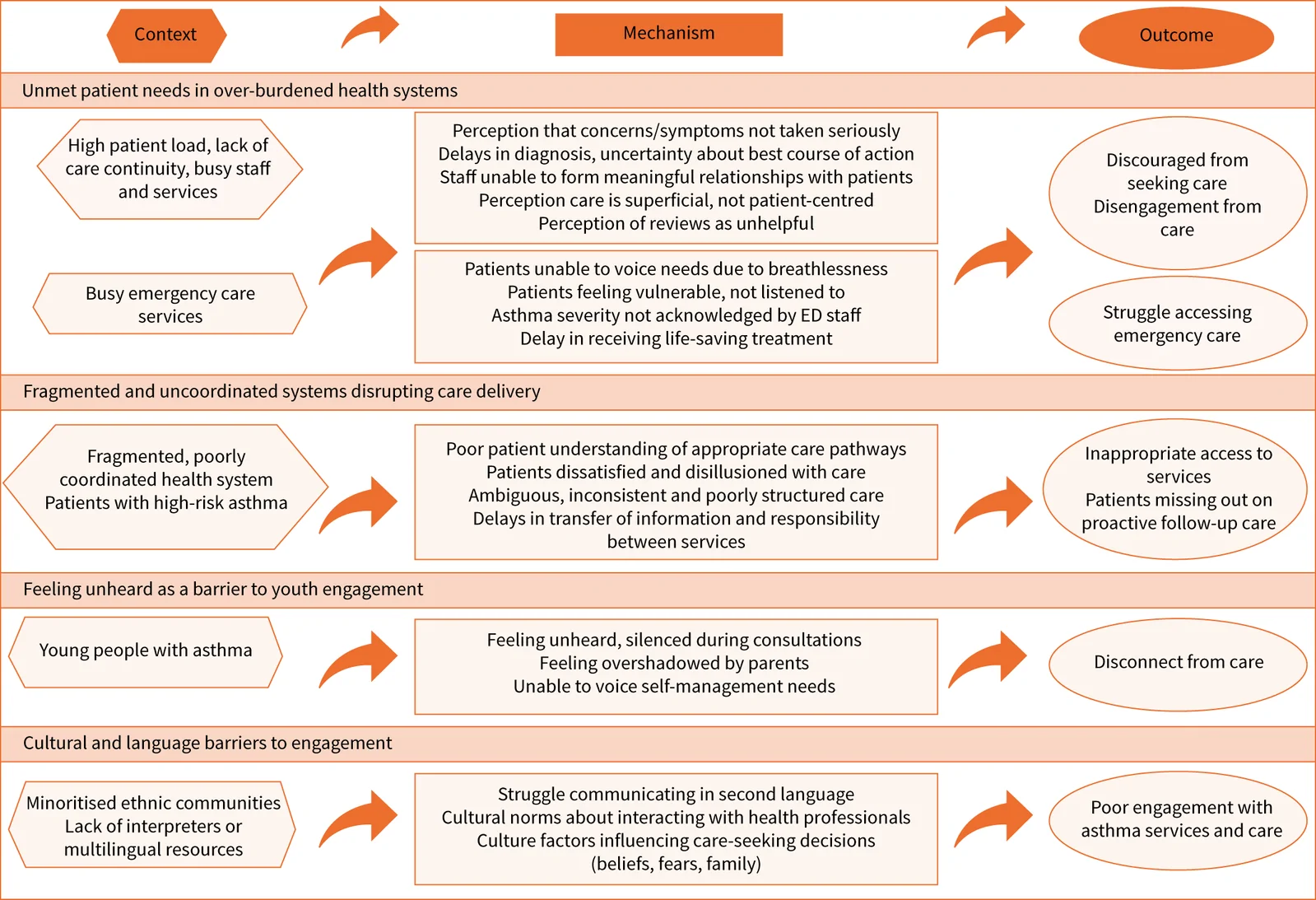
Systemic barriers. ED: emergency department.

#### Theory 5: unmet patient needs in over-burdened health systems

*In overburdened health systems (context), patients with asthma may disengage from care (outcome) due to unmet care needs, such as not being taken seriously by professionals, a lack of patient-centred care and an inability to voice their needs (mechanisms).*

Six articles [36–39, 42, 44] contributed data to this theory. Evidence suggests that, when health systems are overstretched due to high patient loads, patients are discouraged from seeking care, as the care they receive does not fully meet their needs. Lakhanpaul *et al*. [37–39] and Hudson *et al*. [36] reported that patients felt their concerns were not taken seriously by professionals or were reluctant to diagnose a child with asthma, which led to delays in receiving appropriate care. Van Baar *et al.* [42] stated that a lack of continuity of care in outpatient services meant that patients were unable to form meaningful relationships with healthcare providers, leading to the perception of care as superficial and not patient-centred. The ERG agreed that nonattendance at regular reviews could be due to patients finding reviews “unhelpful” if their needs were not met, thus leading to nonattendance. Similar mechanisms were observed in the emergency care context, where Newell *et al*. [44] observed that patients with severe asthma struggled to access emergency help as they felt unable to voice their needs while having an exacerbation, leading them to feel vulnerable, afraid and unheard.

*Supported self-management training for professionals or tools such as asthma passports (interventions) can improve communication, foster more patient-centred care (mechanisms) and encourage timely and sustained patient engagement (outcome).*

Training healthcare professionals in supported self-management may encourage sustained engagement. In their observational study of routine primary care asthma consultations, Kinley *et al*. [45] found that supported self-management training enhances practitioners’ ability to use consultation time, whether in-person or remotely, more effectively, enabling greater focus on discussion of self-management strategies and behaviour change, resulting in higher levels of patient-centred care. In an emergency care setting, interventions could facilitate communication of patients’ needs to emergency care staff during an acute asthma attack. Newell *et al*. [44] co-designed an “asthma patient passport” that provides emergency information, records relevant clinical and demographic information and details unique signs and symptoms, triggers, and previously effective treatments. This passport was found to improve patients’ likelihood of accessing emergency care in a timely manner, as it enabled communication to staff at a time when they were too breathless to talk.

#### Theory 6: fragmented and uncoordinated systems disrupting care delivery

*When healthcare systems are fragmented and poorly coordinated (context), patients with asthma often experience confusion, inconsistent advice and delays in care (mechanisms), leading to inappropriate service use and missed opportunities for proactive management (outcomes).*

Eight articles [30, 31, 36–39, 41, 42] supported the theory that inefficiencies at the level of health systems, such as fragmented care or poor coordination, resulted in poor access to and engagement with care at the patient level. Complex, fragmented systems lead to poor understanding among patients of appropriate care pathways and how to access them [30]. Poorly organised clinics and long waiting times leave patients dissatisfied and disillusioned with care due to a lack of confidentiality and the risk of infection [42]. In poorly coordinated systems, patients report receiving ambiguous, inconsistent and poorly structured care from different clinicians, leading to confusion and inappropriate access to health services [30, 36–39, 41]. Poor care coordination leads to delays in the transfer of clinical information and responsibility between services and a lack of prioritisation of high-risk patients, resulting in patients missing out on proactive follow-up care [31].

*Implementing integrated, multidisciplinary care with clear roles, better communication and partnerships (intervention) can streamline care delivery, improve asthma control (mechanism) and ensure that high-risk patients receive timely preventive support (outcome).*

Two intervention studies targeted systemic barriers to improve outcomes for patients with poorly controlled, high-risk asthma. Fawcett *et al*. [31] implemented an integrated, multidisciplinary team approach, which included a named healthcare professional and partnerships with trained school nurses. This approach improved care coordination, communication and information sharing between different services (*e.g.* primary care and schools). Kercsmar *et al*. [46] implemented a system-wide care coordination programme spanning community, outpatient and inpatient settings, which facilitated increased screening, greater use of asthma action plans and timely and appropriate referrals. Both programmes enhanced partnership working with school nurses, extending the reach of primary care services to children with asthma.

#### Theory 7: feeling unheard as a barrier to youth engagement

*Young people with asthma (context) often feel unheard during consultations (mechanism), which can disconnect them from care and limit their ability to express self-management needs (outcome).*

Support for this theory came from a systematic review by McTague *et al*. [47] of 18 qualitative studies exploring young people's experiences of asthma self-management education. The review highlighted that young people feel “divorced” from healthcare consultations and overshadowed by parents during consultations, limiting their ability to voice their self-management needs.

*Pre-consultation tools and digital support, such as symptom trackers and action plans (intervention), can empower patients to participate more actively in their care (outcome) by building confidence, prompting meaningful dialogue and enhancing communication with healthcare teams (mechanism).*

The review by McTague *et al*. [47] identified potential solutions to address limited engagement among young people in asthma reviews. Pre-consultation aids or communication tools were suggested, as these allowed patients time to think of relevant questions prior to the consultation and acted as prompts, allowing patients to seek out relevant information from care providers. Symptom trackers or monitoring tools were considered acceptable and useful by young people as they provided accessible and real-time support, thereby enabling growth in confidence and autonomy in disease management. Digital asthma action plans were preferred as they can provide specific management instructions based on real-time symptoms and timely communication with healthcare teams, further enhancing responsiveness and continuity of care. The ERG indicated that digital tools (such as apps, decision support tools and symptom trackers) could improve engagement among all patient groups and may be particularly useful for engaging high-risk, nonengaging patients, which is supported by recent literature [48].

#### Theory 8: cultural and language barriers to engagement

*When health systems do not offer options to communicate with patients in forms they can understand or that are culturally relevant (e.g. provide interpreters, translated materials or employing a workforce with similar culture background to their patients) (context), patients or carers from minority ethnic communities may not engage in healthcare (outcome) because they do not understand information shared with them or feel excluded from seeking care (mechanism).*

Six articles [30, 31, 36–39] explained barriers to poor access and engagement among patients and carers from ethnic minority communities. First was the language barrier. Buetow *et al*. [30] and Fawcett *et al*. [31] found that, when interpreter services or multilingual resources were lacking, patients who struggle to communicate in English were less likely to attend review appointments as their ability to understand and communicate asthma-related information was limited. Hudson *et al*. [36] and Lakhanpaul *et al*. [37–39] reported cultural differences in factors influencing decisions about seeking care, which were also noted as barriers. Buetow *et al*. [30] observed that an apparent low engagement with care among patients from minority ethnic groups was due to cultural norms around interacting with authority figures and beliefs or fears associated with healthcare.

## Discussion

### Summary of findings

We developed eight theories proposing mechanisms that shape access to and engagement with asthma services and the contexts that can trigger these mechanisms. Our findings provide nuanced insights into the factors that facilitate or hinder access to and engagement with asthma services, highlighting potential targets for interventions to improve care delivery and patient outcomes.

Overall, the findings suggest that, at the individual level, socioeconomic disadvantage, uncertain candidacy for asthma care, low perceived value of regular follow-up and a disconnect between perceived and actual asthma control limit routine engagement with care. Conversely, supportive patient–provider relationships and convenient alternatives such as telephone reviews can enhance access. At the system level, over-burdened, fragmented and poorly coordinated services reduce patient trust and continuity, particularly among high-risk groups, young people and those from minority ethnic backgrounds. Interventions that reduce structural barriers, facilitate timely follow-up, provide valued and proactive reviews, strengthen relationships, and improve cultural sensitivity and care coordination are most likely to improve sustained engagement and asthma outcomes (see table 2).

**TABLE 2:** Implications for patients, HCPs and practice and policy: potential targeted interventions and their likely mechanisms to improve access and engagement with asthma care

Implications for patients, HCPs, practice and policy	Possible effective intervention strategies to improve access and engagement in asthma care/services	Possible mechanisms of action
**Patients with asthma**	Use of remote tools/telephone for increased convenience	Remove reliance on patient initiative No need for transport/costs No loss of wages/missed work Enable prioritisation of asthma management
** **	Use of digital tools such as pre-consultation aids, symptom trackers and digital asthma action plans	Sufficient time to think of relevant questions to ask during consultation Prompts during consultation Accessible and real-time support Increased confidence and autonomy in disease management Specific management instructions based on real-time symptoms Timely communication with HCPs
**HCPs supporting patients living with asthma**	Delivery of supported self-management	Greater focus on discussion of self-management strategies and behaviour change → increased patient-centred care (meeting patient needs) Patients more able to recognise worsening symptoms and seek timely help Improved patient perception of candidacy for care
** **	HCPs to arrange follow-up asthma reviews immediately after the experience of exacerbations and emergency care use	Patients more receptive to self-management education Increased patient motivation to improve self-management Improved skills in symptom recognition and asthma control
** **	Formalised follow-up through telephone reviews or pharmacist-initiated GP referrals	Enhanced access to proactive care More goal-oriented/focused discussions Increased perception of value of routine care Enhanced patient awareness of susceptibility to exacerbations More therapy changes by GPs
** **	HCPs to contact and question patients about their nonengagement	Increased sense of obligation motivated by a desire to maintain positive relationship with the care providers Increased perception of genuine provider concern about their care
** **	HCPs to provide culturally relevant asthma reviews/care	Improved communication and engagement in care
**Practice and policy**	Provision of training for HCPs in supported self-management	Improved HCP ability to use consultation time more effectively
** **	Development of resources, *e.g.* asthma patient passport Interpreters, culturally sensitive information/resources	Better communication of patient needs when too breathless to talk Enhanced feelings of being listened to and supported Smoother flow through care Improved communication and engagement in care
** **	Improvements in integrated, multidisciplinary and coordinated team approaches	Improved care coordination, communication and information sharing between different services Greater use of asthma action plans
** **	Enhanced training and partnership working with school nurses	Increased asthma screening in schools Timely and appropriate referrals Increased reach of primary care to children with asthma

GP: general practitioner; HCP: healthcare professional.

### Interpretation of findings in relation to current literature

This RRR is the first review that has explored the multi-level factors influencing people's access to and engagement with asthma services. Other parallel work from the PACE programme [49, 50] has identified that the burden of asthma, in terms of control and risk of attacks, varies and is a major factor in determining engagement with routine asthma care. Nonengagement can be predicted, and findings from qualitative research suggest that a targeted approach would enable nonengagement to be addressed proactively in patients with poor asthma control [49, 50]. Our findings align with and build on the conceptual framework of access developed by Levesque *et al*. [25], which emphasised the importance of considering both patient- and supply-side factors in the context of access to asthma care, highlighting that, for some patients, available care (*e.g.* asthma reviews) did not meet their needs. We also drew on the mid-range theory of candidacy [51], which resonated strongly with the data, indicating that patients and their families were often aware of symptoms but did not feel they had the right to seek care.

The association between low income and an increased risk of asthma exacerbations in both children and adults is well documented [16–19]. The mechanisms for this association were described as the psychological burden and financial instability associated with unemployment, which hinders effective self-management, leading to individuals delaying or forgoing healthcare-seeking behaviours and ultimately presenting to emergency departments (ED) with more severe symptoms [19]. An alternative insight potentially reinforcing this behaviour was a perception that EDs offer increased expertise and knowledge [36–40]. The most deprived groups similarly have more emergency hospital admissions and a substantially higher risk of death, highlighting the urgent need for interventions designed specifically to improve treatment and outcomes for disadvantaged groups [5, 52]. Our understanding (Theory 1) posits that competing demands and insurmountable constraints on time and resources limit the capacity of those from disadvantaged socioeconomic backgrounds to access care, but mechanisms for this association are multifaceted and “yet to be resolved” [16]. The inverse care law (“the availability of good medical care tends to vary inversely with the need for it in the population served” [53, 54]) is likely to be a major factor, as people with chronic respiratory diseases from socioeconomically deprived communities receive poorer care [16, 19].

Aligned with other research [55], we demonstrated how misaligned perceptions between patients and providers undermine engagement in asthma care. Providers’ limited awareness of patients’ financial pressures, lifestyle constraints and adherence challenges echo the socioeconomic and structural barriers highlighted in Theory 1. Observed discrepancies in treatment priorities [55] mirror our findings on low perceived or unmet needs and uncertainty about when to seek care (Theories 2 and 5). Taken together, the evidence emphasises the central importance of patient-centred communication, shared understanding and contextualised support as foundations for improving access, sustained engagement and outcomes in asthma care.

Our findings resonate with existing research that has explored the use of the ED as an opportunity for optimising long-term management [56, 57]. Acute care encounters often represent missed opportunities to influence long-term asthma management. Clinicians (understandably) prioritise stabilisation of the acute situation over preventive or educational interventions, reflecting a belief that the ED is not the “right place” for long-term management conversations [57]. This mirrors the theories identified in this review of uncertain candidacy, fragmented care pathways and unmet patient needs, where patients cycle between emergency and routine care without receiving the self-management support or relational engagement needed to break patterns of recurrent exacerbations. Within UK primary care, GPs and nurses have different roles [58]. GPs provide acute management whilst routine reviews of stable asthma are usually undertaken by nurses, typically guided by a computer template that prompts the expected components of a review. The observed association between attending nurse-led asthma clinics and better asthma control [41], however, is complex. Clinics allocate time for attendees to receive a structured review, which may improve outcomes. Conversely, patients selecting to attend an asthma review clinic may be more likely to adhere to routine care (including regular medication) than those who seek unscheduled care from their GP.

The findings highlight the need for more structured, integrated approaches that link acute care to proactive follow-up, strengthen communication and information sharing across settings, and ensure that acute presentations trigger timely educational support aimed at improving long-term asthma control.

### Strengths and limitations

A key strength of this review is the use of RRR methodology, which allowed the integration of empirical evidence with patient and professional perspectives to develop contextually grounded explanatory theories. The inclusion of multiple international study settings and populations strengthens the transferability of findings across comparable healthcare systems and contexts.

However, limitations include a weakness of realist methodology, namely the subjectivity of the data extraction process and the challenge of extracting unbiased CMOs, which may have affected the robustness of some CMO configurations and theory development. To address this limitation, we ensured that all relevant contextual information exploring access and engagement with asthma services was included. An important strength was the multidisciplinary ERG, which supported comprehensive data identification, added rigour to theory development and grounded the interpretation and implications in the context of the NHS. This was a validation role: all conclusions were based on the literature reviewed, not solely on ERG input.

### Implications for future research, practice and policy

Table 2 lists potentially effective interventions and summarises the likely mechanisms by which they could improve access to and engagement with asthma care. This will allow clinicians, policymakers and researchers to identify strategies likely to influence the mechanisms they wish to target and design interventions suited to their individual context.

Strategies to improve access and engagement require proactive, timely, formalised and accessible follow-up, improved patient–professional communication, integrated and coordinated multidisciplinary care, supported self-management, and culturally sensitive resources. For patients, strategies aiming to improve access and engagement with asthma care and tackle health inequalities include the use of remote tools and telephone consultations for increased convenience and digital tools for young people. Interventions to support healthcare professionals include supported self-management, proactive follow-up and patient contact, and the provision of culturally relevant asthma reviews and care. Recommendations for practice and policy include training healthcare professionals in supported self-management, developing culturally sensitive resources for patients, improving integrated, multidisciplinary and coordinated team approaches, and enhancing training and partnership working with school nurses. Further research involving broader patient populations could enhance understanding of context-specific mechanisms and intervention effectiveness.

## Conclusion

We have elucidated important mechanisms and identified potential strategies to address barriers to accessing asthma care that can be tailored to variations in asthma symptoms, triggers and risks, as well as patients’ circumstances. The use of digital tools, patient education, skill development and tools, proactive follow-up after nonattendance, training for healthcare professionals, information sharing between services, and enhanced professional–patient relationships are strategies that could be targeted to improve patients’ access to and engagement with asthma care.

**Points for clinical practice**

- Healthcare professionals need to be proactive and flexible in their approach to engaging patients for asthma care by offering remote or telephone consultations, asking about and (where possible) responding to reasons for missed appointments, and providing digital healthcare options to facilitate communication.
- The perceived value of each consultation can be increased by providing tailored education, defining clear escalation plans, supporting self-management and offering digital options to empower people with asthma (particularly young people).
- Tackling systemic barriers, reducing fragmentation and enabling data sharing across settings could improve individual patients’ access to and engagement with asthma care.

## Supplementary material

**Please note:** supplementary material is not edited by the Editorial Office, and is uploaded as it has been supplied by the author. Supplementary material ERR-0042-2026.SUPPLEMENT
