# Supplementary material for: The context and mechanisms that contribute to accessible and engaging asthma care: a rapid realist review
Source: Eur Respir Rev. 2026 Nov 4;35(182):260042. doi: 10.1183/16000617.0042-2026 (PMC13770365; doi:10.1183/16000617.0042-2026)
Supplement: Supplementary file 1 [file ERR-0042-2026.SUPPLEMENT.pdf]

Supplementary materials

1: Search Strategy

APA PsycInfo <1806 to November 2024 Week 2> Embase Classic+Embase <1947 to 2024 November 14 > Ovid MEDLINE(R) ALL <1946 to November 14, 2024>

#	Query	Results<br>15 Nov. 24
1	*"Referral and Consultation"/ or *professional referral/ or *client transfer/ or *self-referral/ or *patient referral/ or *referral visit/	56,944
2	*Access to Primary Care/ or *Health Services Accessibility/ or *access to information/ or *access to medication/ or *access to treatment/ or *health care access/ or *"Healthcare Access and Quality Index"/ or *Health Care Utilization/	145,307
3	*engagement/ or *consumer engagement/ or *patient engagement/ or *social engagement/ or *disengagement/	20,784
4	*hospital readmission/	31,883
5	or/1-4	252,626
6	Asthma/ or Asthma-Chronic Obstructive Pulmonary Disease Overlap Syndrome/ or (asthma$ or wheez$).ti. or wheez$.ti,ab. or (asthma$ or wheez$).ti,ab. or Bronchial Hyperreactivity/ or bronchial$ hyperreactivit$.ti,ab. or allergic asthma/ or Asthma, Aspirin-Induced/ or aspirin exacerbated respiratory disease/ or Cough-Variant Asthma/ or cough variant asthma/ or eosinophilic asthma/ or Asthma, Exercise-Induced/ or exercise induced asthma/ or extrinsic asthma/ or intrinsic asthma/ or mild intermittent asthma/ or mild persistent asthma/ or moderate persistent asthma/ or nocturnal asthma/ or Asthma, Occupational/ or occupational asthma/ or severe asthma/ or thunderstorm asthma/ or severe persistent asthma/ or Dyspnea/ or Dyspnea, Paroxysmal/ or (asthmatic? or (asthma$ adj3 (sever$ or chronic$ or patient? or primary or major))).ab.	888,756
7	pyroglyphidae/ or dermatophagoides farinae/ or dermatophagoides pteronyssinus/ or (pollution or pollutant? or dust? or mites).ti. or Air Pollution/ or Air Pollution, Indoor/ or Tobacco Smoke Pollution/ or air pollutant/ or Risk Factors/ or Ecological Factors/ or Social Ecology/ or Environmental Effects/	2,613,500
8	8 and (Somatoform Disorders/ or Psychosomatic Medicine/ or	14,502

	Antigens/ or dust/)	
9	(asthma adj5 program$).ti.	2,075
10	or/6-9	3,427,887
11	United Kingdom/ or Aruba/ or Albania/ or Andorra/ or United Arab Emirates/ or Argentina/ or Armenia/ or American Samoa/ or "Antigua and Barbuda"/ or Australia/ or Austria/ or Azerbaijan/ or Belgium/ or Bulgaria/ or Bahrain/ or Bahamas/ or "Bosnia and Herzegovina"/ or Belarus/ or Belize/ or Bermuda/ or Brazil/ or Barbados/ or Botswana/ or Brunei/ or Canada/ or Switzerland/ or Channel Islands/ or Chile/ or China/ or Colombia/ or Costa Rica/ or Cuba/ or Curacao/ or Cayman Islands/ or Cyprus/ or Czech Republic/ or Germany/ or Dominica/ or Denmark/ or Dominican Republic/ or Ecuador/ or Spain/ or Estonia/ or Finland/ or Fiji/ or France/ or Faroe Islands/ or Gabon/ or United Kingdom/ or Georgia/ or Gibraltar/ or Equatorial Guinea/ or Greece/ or Grenada/ or Greenland/ or Guatemala/ or Guam/ or Guyana/ or Hong Kong/ or Croatia/ or Hungary/ or Indonesia/ or Isle of Man/ or Ireland/ or Iraq/ or Iceland/ or Israel/ or Italy/ or Jamaica/ or Japan/ or Kazakhstan/ or "Saint Kitts and Nevis"/ or "Republic of Korea"/ or Kuwait/ or Libya/ or Saint Lucia/ or Liechtenstein/ or Lithuania/ or Luxembourg/ or Latvia/ or Macau/ or Monaco/ or Moldova/ or Maldives/ or Mexico/ or Marshall Islands/ or North Macedonia/ or Malta/ or Montenegro/ or Northern Mariana Islands/ or Mauritius/ or Malaysia/ or Namibia/ or New Caledonia/ or Netherlands/ or Norway/ or Nauru/ or New Zealand/ or Oman/ or Panama/ or Peru/ or Palau/ or Poland/ or Puerto Rico/ or Portugal/ or Paraguay/ or French Polynesia/ or Qatar/ or Romania/ or Russia/ or Saudi Arabia/ or Singapore/ or El Salvador/ or San Marino/ or Serbia/ or Suriname/ or Slovak Republic/ or Slovenia/ or Sweden/ or Sint Maarten/ or Seychelles/ or Thailand/ or Turkmenistan/ or Tonga/ or "Trinidad and Tobago"/ or Turkey/ or Tuvalu/ or Taiwan/ or Uruguay/ or United States/ or "Saint Vincent and the Grenadines"/ or British Virgin Islands/ or United States Virgin Islands/ or Kosovo/ or South Africa/ or Venezuela/	8,193,033
12	((("St. Martin" or "the Collectivity of Saint Martin") and ("fran$" or "french")) or ("West Bank" or "Gaza") or "Turks and Caicos Islands").mp. or developed countries/	65,867
13	11 or 12	8,223,585
14	("health care need*" or "healthcare need*" or "patient?? needs").ab,ti,sy,kf,kw,tm,hw,ox,tw,od,id,ec,xs,mh,fs,kw,sh,dj,cc. or	160,151

	needs.id,kf,od,mf,cw.	
15	("health care need*" or "healthcare need*" or "patient?? needs").ab,ti,sy,kf,kw,tm,hw,ox,tw,od,id,ec,xs,mh,fs,kw,sh,dj,cc. or needs.id,kf,od,mf,cw. or *Biological Markers/ or *Early Diagnosis/ or *Early Intervention/	393,079
16	("perception of need*" or (percei* adj2 need*)).ab,ti,sy,kf,kw,tm,hw,ox,tw,od,id,ec,xs,mh,fs,kw,sh,dj,cc.	20,379
17	(("desire for" adj1 care) or ("desire for" adj1 healthcare)).ab,ti,sy,kf,kw,tm,hw,ox,tw,od,id,ec,xs,mh,fs,kw,sh,dj,cc.	71
18	("health care seeking" or "healthcare seeking" or "care seeking").ab,ti,sy,kf,kw,tm,hw,ox,tw,od,id,ec,xs,mh,fs,kw,sh,dj,cc.	20,764
19	((("health care" or "healthcare" or "care") adj1 "reach*") or (("health care" or "healthcare" or care or clinic) adj2 ("use" or using or utili* or attend*))).ab,ti,sy,kf,kw,tm,hw,ox,tw,od,id,ec,xs,mh,fs,kw,sh,dj,cc.	324,471
20	*Patient Acceptance of Health Care/ or *"Treatment Adherence and Compliance"/ or *Patient Compliance/ or (Patient Accept* adj2 (Therapy or Intervention or Treatment or "Health Care" or Healthcare)).ti,ab,id,kf,od,mf,cw.	120,794
21	or/14-20	846,890
22	10 and 13 and 5	6,208
23	22 and 21	2,056

Cochrane Library <no limits>

#	Query	**Results**<br>**15 Nov. 24**
1	[mh ^asthma] OR [mh ^"Asthma-Chronic Obstructive Pulmonary Disease Overlap Syndrome"] OR (asthma* OR wheez*):ti OR (wheez*):ti,ab or (asthma* OR wheez*):ti,ab OR [mh ^"Bronchial Hyperreactivity"] OR (bronchial* hyperreactivit*):ti,ab OR [mh ^"allergic asthma"] OR [mh ^"Asthma, Aspirin-Induced"] OR [mh ^"aspirin exacerbated respiratory disease"] OR [mh ^"Cough-Variant Asthmas"] OR [mh ^"Cough Variant Asthma"] OR [mh ^"Asthma, Cough-Variant"] OR [mh ^"eosinophilic asthma"] OR [mh ^"Asthma, Exercise-Induced"] OR [mh ^"exercise induced asthma"] OR [mh ^"extrinsic asthma"] OR [mh ^"intrinsic asthma"] OR [mh ^"mild intermittent asthma"] OR [mh ^"mild persistent asthma"] OR	41,292

	[mh ^"moderate persistent asthma"] OR [mh ^"nocturnal asthma"] OR [mh ^"Asthma, Occupational"] OR [mh ^"occupational asthma"] OR [mh ^"severe asthma"] OR [mh ^"thunderstorm asthma"] OR [mh ^"severe persistent asthma"] OR [mh ^"Dyspnea"] OR [mh ^"Dyspnea, Paroxysmal"] OR (asthmatic? OR (asthma* NEAR/3 (sever* or chronic* or patient? or primary or major))):ab OR [mh ^"Antigens, Dermatophagoides" [mj]] OR [mh "Somatoform Disorders" [mj]]	
2	([mh dust] or [mh Antigens] or [mh pyroglyphidae] or [mh "Air Pollution"] or [mh "Air Pollution, Indoor"] or [mh "Air Pollutants"] or [mh "Tobacco Smoke Pollution"] or (pollution or pollutant?):ti,ab,kw or [mh "Ecological and Environmental Phenomena" [mj]] or [mh "Social Environment" [mj]]) AND #1	1,954
3	#1 or #2	41,292
4	(("health care" NEXT need*) OR (healthcare NEXT need*) OR (patient?? NEXT needs) OR needs OR ("perception of" NEXT need*) OR (percei* NEAR/2 need*) OR ("desire for" NEAR/1 care) OR ("desire for" NEAR/1 healthcare) OR ("health" NEXT "care seeking") or "healthcare seeking" or "care seeking" OR ((("health care" or "healthcare" or "care") NEAR/1 reach*) OR (("health care" or healthcare or care or "clinic") NEAR/2 (use or using or utili* or attend*)))):ti,ab,kw OR ([mh ^"Patient Acceptance of Health Care" [mj]] OR [mh ^"Treatment Adherence and Compliance" [mj]] OR [mh ^"Patient Compliance" [mj]])	48,945
5	[mh "Early Medical Intervention"] OR [mh "Internet-Based Intervention"] OR "Risk Factors" OR ([mh ^bioMarkers [mj]] OR [mh ^"Early Diagnosis" [mj]] OR [mh ^"Early Intervention, Educational" [mj]])	75,390
6	(#3 and #4 and #5) NOT ((embase):an)	43 –<br><br>(30 Trials, 13 Reviews)

Supplementary Materials 2: Characteristics and CMOCs extracted from included studies

Author, Year, Country	Study Aim & Setting	Design and methods	Context-Mechanism-Outcome Configurations (CMOCs)	Supports theory number
**Buetow et al, 2002**<br>New Zealand	Describe and explain poor patient understanding of when and how to access GP care for childhood asthma.<br><br>Primary care	Qualitative.<br><br>Semi-structured interviews with 'Key informants' representing provider and managerial perspectives (n=29).<br><br>Mixed ethnic group (Maori, White NZ, Samoan, Togan)	1. If language barriers are present and patients are not provided access to care in their own language (e.g. through interpreters, adapted or inclusive resources) (C), this limits patients' ability to understand their asthma and its management (M), which results in poor patient engagement (O).<br>2. If patients have low-education levels (C), they have limited ability to understand health related information (e.g. underestimate severity, incorrect health beliefs) (M), which makes them less likely to access and engage in care (O).<br>3. If care is less well coordinated (C), patients receive different messages from different providers/HCPs, leading to confusion (M), which then results in poor engagement (O).<br>4. If the healthcare system is complex (e.g. no clear pathway?) (C), patients may have poor understanding of when or how to seek primary (GP) care (M), which results in poor access and engagement in care (O).<br>5. If patients come from different cultural backgrounds (C), they may act with humility and respect, in a low position of honour (M), which may appear as low engagement in care (O).<br>6. Patients from minority ethnic populations (in this case, pacific and islanders) (C), may be discouraged from seeking care (O), due to fears associated with GP care (e.g. fear of diagnosis, treatment, causing/receiving offence, wasting GP time) or unhealthy optimism (e.g. thinking the problem will resolve on its own) (M).	1, 6, 8
**Van Baar et al, 2006**<br>UK	Understand factors influencing patients' decisions to attend for outpatient follow up consultations for asthma; explore	Qualitative.<br>Interviews with patients with moderate to severe asthma (n=19).	1. Patients who are motivated to monitor or improve their disease control (C), are more likely to attend review appointments (O), often because they see asthma review as an opportunity to better understand and control their asthma, benefit from recent therapeutical advances and do not wish to jeopardize the valued relationship with their doctor (Ms).	4, 5, 6,

Author, Year, Country	Study Aim & Setting	Design and methods	Context-Mechanism-Outcome Configurations (CMOCs)	Supports theory number
	attitudes to telephone and email consultations<br><br>Secondary care (hospital outpatient clinic)	Mix of attenders and non-attenders; (>60 White)	2. In hospital outpatient services with long waiting times and poorly organized clinics (C), patients have concerns about lack of confidentiality and risk of infections, which lead to disillusionment and dissatisfaction with care (M), which is a common reason for non-attendance at reviews (O).<br>3. In hospital outpatient services where there's lack of continuity of care (C), patients find it difficult to develop meaningful relationships with HCPs and feel HCPs provide superficial rather than holistic care (e.g. do not go into route of the problem) (Ms), which discourages future attendance at reviews (O).<br>4. If asthma reviews were delivered by telephone (C), this will minimize travel and waiting times (M), which may increase access and attendance to asthma reviews (O), but only if an option of switching to face-to-face review was available when needed (M).	
**Kinley et al 2024 UK**	Understand how health professionals deliver supported self-management in UK clinical practice. Explore whether mode of review or implementation support influence delivery.<br><br>Primary care	Observational. Video recordings of F2F and telephone asthma reviews conducted by 12 professionals trained in supported self-management (n=64).	1. In busy UK primary care services characterized by high time pressures (C), training healthcare professionals in supported self-management and behaviour change (I) leads to enhanced skills in effectively using consultation time to include more discussion of self-management strategies and behaviour change (M), which in turn leads to increased delivery of patient-centred care (thus increased patient engagement in care) (O)<br>2. When delivering remote consultations/asthma reviews (C), training healthcare professionals in supported self-management and behaviour change (I), leads to similar increase in discussion of self-management strategies and behaviour change as in face-to-face reviews (M), which in turn leads to increased delivery of patient-centred care (thus increased patient engagement in care) (O)	5
**Coffey et al 2012 USA**	Describe lived experience of Puerto Rican families of children with asthma and their use of ED	Qualitative. Interviews with mothers referred to asthma centre as a result of an ED visit	1. Parents of children with asthma (C), access or engage in emergency healthcare (O) when they perceive their child's symptoms worsening or when the action plans do not seem to work (M).<br>2. Parents of children with asthma (C), access or engage in emergency healthcare (O) because they believe asthma care is better in hospital	2

Author, Year, Country	Study Aim & Setting	Design and methods	Context-Mechanism-Outcome Configurations (CMOCs)	Supports theory number
	for asthma care.<br><br>Emergency care	(n=10).	compared to the primary care clinic (M).	
**Daines et al 2020 UK**	Explore how patients develop and establish their self-management strategies and how health professionals could best support the process.<br><br>Primary care	Qualitative. Interviews and focus groups with adults and parents of children with asthma (n=49).	1. Patients with traumatic past experiences with their asthma (C), tend to seek /access professional help (O) because they feel less confident about managing their asthma (lower perception of control) (M).<br>2. Patients living with asthma for a long period of time (C), develop self-management skills and strategies over time (O), through a mixture of experiential learning and input from HCPs (M)<br>3. In the context of asthma care (C), arranging asthma consultations around the time patients have experienced asthma symptoms (I), may lead patients to be more receptive to support (M), resulting in better engagement (O).	2, 4
**McDonough & Mault, 2013 UK**	Explore patients' reasons for failing to attend clinics for difficult asthma. Evaluate whether telephone contact after missed appointment affected future attendance.<br><br>Secondary care (hospital clinic)	Quantitative. Telephone survey of nonattenders to explore reasons and asthma control (n=51).<br><br>Review of electronic records of attendance.	1. Patients with difficult asthma (complex care needs) (C), fail to attend asthma reviews (O) because of memory lapses or forgetting (M)<br>2. Patients with difficult asthma (complex care needs) (C), when contacted/questioned about their previous non-attendance (I) are more likely to attend future asthma review appointments (O), possibly because not wanting to let the doctor down or feeling doctors are genuinely interested in their care (M)	4
**Lakhanpaul et al, 2014, 2017, 2020. Hudson et**	Explore experiences of asthma and asthma management among	Qualitative. Focus groups with South Asian families (n=63);	1. Parents of south Asian children living in the UK (but common to all communities) (C), express confusion and uncertainty about symptoms, causes and management of asthma which impedes their ability to recognize their children's symptoms as requiring medical	2,5,6,8

Author, Year, Country	Study Aim & Setting	Design and methods	Context-Mechanism-Outcome Configurations (CMOCs)	Supports theory number
**al 2016. UK**	South Asian and White British families.<br><br>Develop an intervention planning framework for South Asian children with asthma.<br><br>Primary care	Interviews with South Asian and White children (n=47) and carers (n=61) and HCPs.<br><br>Intervention mapping and community-based participatory research (data integration + workshops) to develop intervention (ACT on Asthma)	assistance (i.e. ability to claim candidacy for healthcare) (M), resulting in poor health-seeking (O).<br>2. Parents of south Asian children living in the UK (C), often receive advice and guidance on their children's health from members of their extended family, which may add to the ambiguity around candidacy (recognizing symptoms require help) (M), affecting their access and/or engagement with services (O).<br>3. Parents of south Asian children living in the UK (C), tended to overcome the language related barriers to healthcare (O) by seeking out healthcare professionals (GPs, pharmacists) who were from same cultural and linguistic background or offer services in a language other than English (M)<br>4. Parents of South Asian and White children living in the UK (C), felt healthcare professionals not taking their concerns seriously or being reluctant to diagnose child with asthma (M), which lead to delays and difficulties in receiving appropriate diagnosis (further creating uncertainties about the best course of action) and future engagement with services (O).<br>5. Parents of south Asian children living in the UK (C), do not have any formal means of assessing severity of their children's asthma (no action plans, no use of peak flow meters), which often resulted in delays in recognizing the severity of symptoms (M), until ED became the only option, encouraging repeated ED use (O).<br>6. Parents of south Asian children living in the UK (C), who were not accessing annual asthma reviews (O), this was often either because they considered it of little value or had their needs met from other sources (M)<br>7. Parents of South Asian and White children living in the UK (C), report receiving ambiguous advice on symptoms, inadequate explanations about asthma and its treatments and unclear guidance/decisions about management from HCPs (inadequate adjudications) (M), which led to inappropriate access to health services (e.g. repeat visits,	

Author, Year, Country	Study Aim & Setting	Design and methods	Context-Mechanism-Outcome Configurations (CMOCs)	Supports theory number
			second opinions, visits to ED) (O)<br>8. Parents of south Asian children living in the UK (C), often attend to ED more than GP services (O), because of their previous experiences of being referred to ED for acute episodes leading to perception that hospital care is better (more expertise and knowledge about asthma) (M)	
**McTague et al. 2022 UK, USA, Australia, Germany, Sweden, Netherlands**	Identify and synthesise evidence of youths with asthma and their experience of self-management education.	Systematic review and meta-synthesis of qualitative evidence (n=18).	1. Young people (C), often feel unheard and overshadowed by parents in asthma consultations, leading to a feeling of being divorced from healthcare consultations (Ms), which limits their engagement as they have limited opportunities to communicate their self-management education needs (O).<br>2. Young people (C), think that pre-consultation aids or communication tools (I) would help them seek out relevant information during consultations (O) as these would allow time to think of relevant questions and act as prompts during consultations (M)<br>3. Technology that can track/monitor symptoms (I) is found acceptable and useful by young people (C), as it provides greater accessibility to support, thereby enabling a growth in their confidence and autonomy in their disease management (M), which may lead to more active role in management (O).<br>4. Digital asthma action plans (I), are preferred by young people (C) as they can provide specific management instructions based on current reported symptoms (M) as well as provide an opportunity for medical team to contact young people about their condition (M), resulting in better engagement (O).	7
**Newell et al. 2017 UK**	Understand reasons behind patients' reluctance to attend ED<br>Consider practical solutions in a structured way.	Mixed. Soft Systems Method to conceptualise, define and address problems.<br>Cocreation with severe asthma	1. People with severe asthma (C), do not seek emergency medical help (ED) when they need to (O), because they feel vulnerable and afraid, unable to voice their needs when least able to talk, feel not always listened to, feel life-saving treatments are not always escalated and feel HCPs do not appreciate attack's severity (Ms).<br>2. In the context of emergency care (C), implementation of Asthma patient passport (I) improves communication with ED staff and	5

Author, Year, Country	Study Aim & Setting	Design and methods	Context-Mechanism-Outcome Configurations (CMOCs)	Supports theory number
	Emergency care	patients and nurses. Quality improvement approach to test solutions.	facilitates improved access and flow through ED (Ms), resulting in reduced delays and individualized and appropriate treatment (O) and increased likelihood of patients accessing ED when they need to (O).	
**Gruffydd-Jones et al. 1999 UK**	Understand why patients with asthma do not attend for structured nurse-led asthma care in a general practice and describe morbidity characteristics of these patients.<br><br>Primary care	Quantitative. Telephone questionnaire to non-attenders of asthma clinic (n=357).	1. Patients with mild asthma (low morbidity) (C), do not see their asthma as serious enough (M) and hence tend not to attend asthma reviews (O)<br>2. For asthma patients with low morbidity (C), some kind of formalized follow-up is still necessary (I) as it can address and improve the asthma symptoms that this patient group is still likely to experience (M), resulting in better engagement from this group (O).<br>3. Asthma patients with high morbidity who attend GP rather than scheduled reviews (C), may be receiving poorly structured asthma care (e.g. less use of self-management plans, appropriate testing) (M), which results in them missing out on proactive care (O).	3, 6
**Pinnock et al. 2003 UK**	Determine whether routine asthma review by telephone improves access and is good alternative to F2F reviews<br><br>Primary care	Quantitative. Pragmatic RCT; Patients randomised to either telephone or F2F review with asthma nurse. (n=278). Outcome: % of patients reviewed within 3 months of randomisation and QoL.	1. In UK primary care context (C), telephone asthma review consultations (I) improve patients' access to proactive care, without any clinical disadvantage or loss of satisfaction (Os) as they offer acceptable and convenient modes of consultation and more efficient and goal-oriented discussion (Ms).<br>2. Telephone asthma review consultations (I) can improve access to routine care (O) by overcoming the structural barriers to attendance (M).	1, 3
**Fawcett et al 2021**	Understand how young people	Qualitative. Sequential	1. In a system that relies on patients for proactively seeking attention, children from deprived/ethnic minority backgrounds (C), are less likely	1, 6, 8

Author, Year, Country	Study Aim & Setting	Design and methods	Context-Mechanism-Outcome Configurations (CMOCs)	Supports theory number
**UK**	engage with routine asthma services.<br><br>Identify barriers to engagement and potential solutions.<br><br>Primary care	simulation method: a) co-creation of simulation storyboard with patient and professional input, b) simulation workshop with stakeholders (n=37) c)post-simulation discussions.	to access care (O), as their families often have chaotic lifestyles and child's asthma may receive low priority in family worries (Ms)<br>2. Families with language barriers, are less likely to attend review appointments, as they often struggle to conduct reviews in second language (M)<br>3. In overburdened/stretched primary care services, with no formal monitoring of patient attendance (C), GPs lack time and resources to identify patients who may be at high risk (M), leading to such patients being overlooked/missing proactive care (O)<br>4. In services with long waiting times (C), families may default to accessing emergency/unscheduled care (M), which may result in poor engagement in routine, scheduled reviews and missing out on proactive care (O).<br>5. When services are fragmented/uncoordinated (C), there are significant delays in transferring clinical information and responsibility between different teams (M) which may result in patients missing out on proactive and follow up care (O).<br>6. An integrated MDT approach to asthma care with a named HCP (I) may improve care co-ordination, communication, and information sharing (M), which may result in better care planning (O)<br>7. Integration of school nurses (I) within the MDT can be useful as it can facilitate clinicians' access to young people (O), enabling them to deliver education and routine care (M)	
**Serhal et al. 2022 Australia**	Investigate uptake and outcome of pharmacist-initiated GP referrals within a cluster RCT of Pharmacy Asthma Service (PAS)<br><br>Primary/Community	Quantitative. Data on pharmacist-initiated GP referral initiation, uptake and outcomes collected from patients in PAS and usual care arm	1. Patients with poorly controlled asthma, who live in accessible locations and are routine care-seekers (C), a pharmacist-initiated GP referral (I) is effective in increasing the patients' engagement/uptake in care (O), possibly through higher perceived susceptibility about heir vulnerability to impact of asthma (M).<br>2. Patients with poorly controlled asthma referred to GPs by pharmacists (C), were more likely to receive therapy changes by GPs (O) as GPs may perceive the assessment being more in-depth (M) as this was performed by the pharmacists.	1, 3

Author, Year, Country	Study Aim & Setting	Design and methods	Context-Mechanism-Outcome Configurations (CMOCs)	Supports theory number
	care	(n=254).	3. Among patients with pharmacist-initiated GP referral (C), those living in accessible locations (M) were more likely to visit their GP for asthma related consultation (O) than those living in rural and remote localities.	
**Hoffmann et al. 2008 USA**	Identify perceived barriers to follow-up care for adults with asthma. Determine effect of barriers on quality of life and compliance.<br><br>Community healthcare facilities	Quantitative. Survey of adults with asthma accessing community healthcare facilities (n=12).	1. In urban, outpatient, routine healthcare facilities (C), most common patients' barriers to accessing follow-up care (O) were 'long waiting times', 'need to miss work', 'lack of transportation' and 'high cost of care' (M)<br>2. Patients with mild or well controlled asthma (C) deprioritise attending follow-up care for asthma (O), as it necessitates adjusting their work schedules (M).	1, 3
**Kercsmar et al 2017 USA**	Reduce asthma related hospitalisation and ED visits for Medicaid-insured paediatric patients, through an Asthma Improvement Collaborative. Primary & Secondary care	Quantitative. Quality Improvement methods with PDSA cycles testing various inpatient, outpatient and community-based interventions. Outcome: Rates of asthma related hospitalisations and asthma control	1. For children and adolescents with high-risk asthma (C), an improved care coordination program (I) led to increased completion of asthma action plans, asthma control tests, medication delivery (M) resulting in improved patients' asthma control (O)<br>2. Partnership with and training to school nurses (I) extended the reach of primary healthcare services (O) to children with poorly controlled asthma (C), through increased screening of asthma and referrals to appropriate services (M).	6
**Teach et al 2006 USA**	To test whether higher spatial accessibility (SA) to primary care	Quantitative. Secondary analysis of data collected within a	1. Children living in urban areas, predominantly from black and disadvantaged households have (C) lower immediate proximity to sources of paediatric primary care (M), resulting in reduced likelihood of scheduled primary care visits for asthma (O)	1

Author, Year, Country	Study Aim & Setting	Design and methods	Context-Mechanism-Outcome Configurations (CMOCs)	Supports theory number
	paediatric services is associated with more scheduled visits for asthma, better asthma management and fewer unscheduled asthma visits.	RCT of children seeking acute asthma care in ED. Patients' special accessibility to primary care compared with medical management and healthcare utilisation. (n=411)	2. Use of scheduled primary care for asthma is lower (O) among children living in urban, disadvantaged and minority ethnic areas who have lower immediate proximity to sources of primary paediatric care (C).	

Supplementary Materials 3: **Theories developed from 16 studies (19 articles) explaining access/engagement with asthma services**

Context-Mechanism-Outcome-Intervention Configurations	Source articles
**Theme 1: Demographic and social-cognitive barriers**	
**Theory 1**: **Socio-economic and structural barriers to access**<br>Patients from disadvantaged socio-economic backgrounds (C) face multiple structural and contextual barriers, such as low health literacy, financial strain, poor proximity to care, competing life priorities and poor mental health (Ms) that reduce their ability to access and engage in asthma care (O).<br>**1a.** Telephone asthma review consultations (I) can improve access to routine care (O) by overcoming the structural barriers to attendance (M).	Buetow et al 2002; Fawcett et al 2021; Hoffmann et al 2008; Teach et al 2006; Serhal et al. 2022<br><br>Pinnock et al 2003
**Theory 2: Uncertain candidacy and escalation to emergency care**<br>Patients or parents of children with asthma with active symptoms/exacerbations (C), often struggle to recognise when symptoms require medical attention leading to delays in seeking care (M) and defaulting to emergency services once symptoms become severe (O). This uncertainty around candidacy for care, compounded by lack of formal tools to assess severity and past experiences of hospital care, reinforces patterns of emergency over routine care use (O).<br>**2a**: Timely follow-up after exacerbations (I) may increase patient receptiveness (M), improving engagement (O).	Lakhanpaul et al 2014, 2017, 2020; Hudson et al 2016<br>Coffey et al 2012<br><br>Daines et al 2020
**Theory 3: Low perceived need as a barrier to asthma care engagement**<br>Low perception of illness severity (M) among asthma patients (C) can lead to poor engagement with health services (O).<br>**3a.** Formalised follow-up through telephone reviews or pharmacist-initiated GP referrals (I) can enhance patient awareness and perceived value of care (M), resulting in increased accessibility and more proactive management (O).	Gruffydd-Jones et al 1999<br>Hoffman et al 2008<br><br>Serhal et al 2022<br>Pinnock et al 2003
**Theory 4: Relational accountability as a driver of patient engagement**<br>Valued patient/provider relationships and experience of supportive reviews (M) promote engagement in care (O) among patients who understand the importance of self-management (C).<br>**4a.** Follow-up contact from providers after a missed appointment (I) can convey genuine care/concern (M), motivating future engagement (O).	Van Baar et al 2006<br>Daines et al 2020<br><br>McDonough and Mault 2013

Context-Mechanism-Outcome-Intervention Configurations	Source articles
**Theme 2: Systemic Barriers**	
**Theory 5: Unmet patient needs in over-burdened health systems**<br>In overburdened health systems (C), patients with asthma may disengage from care (O) due to unmet care needs such as not being taken seriously by professionals, lack of patient-centred care and inability to voice their needs (Ms).<br>**5a:** Interventions like supported self-management training for professionals or tools like asthma passports (Is) can improve communication, foster more patient-centred care (Ms), and encourage timely and sustained patient engagement (O).	Van Baar et al 2006<br>Lakhanpaul et al 2014, 2017, 2020; Hudson et al 2016; Newell et al 2017<br><br>Kinley et al 2024; Newell et al 2017
**Theory 6: Fragmented and Uncoordinated Systems Disrupting Care Delivery**<br>When healthcare systems are fragmented and poorly coordinated (C), patients with asthma often experience confusion, inconsistent advice, and delays in care (Ms), leading to inappropriate service use and missed opportunities for proactive management (Os).<br>**6a:** Implementing integrated, multidisciplinary care including clear roles, better communication, and partnerships (I) can streamline care delivery, improve asthma control (M), and ensure high-risk patients receive timely, preventive support (O).	Buetow et al 2002; Fawcett et al 2021<br>Lakhanpaul et al 2014, 2017, 2020; Hudson et al 2016 ; Van Baar et al 2006 ; Gruffydd-Jones et al 1999<br><br>Kercsmar et al 2017<br>Fawcett et al 2021
**Theory 7: Feeling unheard as a barrier to youth engagement**<br>Young people with asthma (C) often feel unheard during consultations (M), which can disconnect them from care and limit their ability to express self-management needs (O).<br>**7a.** Pre-consultation tools and digital supports like symptom trackers and action plans (I) can empower patients to participate more actively (O) by building confidence, prompting meaningful dialogue, and enhancing communication with healthcare teams (M)	McTague et al 2022<br><br>McTague et al 2022
**Theory 8: Cultural and language barriers to engagement**<br>Patients or carers from minority ethnic communities (C) may struggle to engage in asthma care (O) due to language barriers and cultural differences that affect communication, understanding, and decision-making (M). Without access to interpreters, culturally sensitive resources, or providers who share their background, these patients may feel excluded or uncertain about seeking care, leading to reduced attendance and engagement in asthma reviews.	Buetow et al 2002<br>Fawcett et al 2021<br>Lakhanpaul et al 2014, 2017, 2020; Hudson et al 2016
